# Colon adenoma and adenocarcinoma with clear cell components - two case reports

**DOI:** 10.1186/s13000-019-0819-z

**Published:** 2019-05-10

**Authors:** Yuzo Oyama, Haruto Nishida, Takahiro Kusaba, Hiroko Kadowaki, Motoki Arakane, Kazuhisa Okamoto, Junpei Wada, Shogo Urabe, Tsutomu Daa

**Affiliations:** 1 Departments of Diagnostic Pathology, Faculty of Medicine, Oita University, 1-1, Idaigaoka, Hasama-machi, Yufu City, 879-5593 Japan; 2 Departments of Gastroenterology, Faculty of Medicine, Oita University, Yufu city, Japan; 3 Division of Clinical Laboratory, Oita Prefectural Hospital, Oita city, Japan

**Keywords:** Colon, Adenocarcinoma, Clear cell change, Electron microscopy

## Abstract

**Background:**

Diagnoses reflect clear cell morphologies when tumor cells have clear cytoplasm in many organs, and the nature of such clear cells is typically identified. Colorectal tubular adenoma or adenocarcinoma, conversely, rarely show clear cells, the reason for which remains uncertain. We report 2 colon tumors with clear cell components (Case 1: adenoma; Case 2: adenocarcinoma) and investigate the nature of the clear cells.

**Case presentation:**

Case 1 was a 75-year-old man with a superficial elevated polyp detected in the rectum for whom endoscopic submucosal dissection was performed. Microscopically, 10% of the tumor showed dysplastic columnar epithelium with clear cytoplasm forming tubular structures accompanied by conventional tubular adenoma. Case 2 was a 58-year-old man with a pedunculated polyp found in his sigmoid colon for which polypectomy was performed. Microscopically, 90% of the tumor showed dysplastic columnar epithelium with clear cytoplasm forming fused glands or cribriform structures adjacent to the ordinal tubular adenocarcinoma. In both cases, clear and ordinary tumor cells were negative for CK7 and positive for CK20 and CDX2, consistent with findings of colorectal origin. Different results were found for CEA and CD10 staining. CEA was positive on the luminal side of the conventional area in contrast diffuse cytoplasmic staining of the clear cell area in both cases. CD10 was only positive for the clear cell component of case 2. The clear cell components were negative for Periodic acid-Schiff (PAS), Alcian blue, and mucicarmine staining and AFP immunohistochemistry. An ultrastructural examination found multiple cytoplasmic lipid-like vacuoles in the clear cell component that were predominantly negative for adipophilin by immunoelectron microscopy.

**Conclusions:**

We investigated tubular adenoma and tubular adenocarcinoma with clear cell components. The accompanying conventional tubular adenoma or adenocarcinoma cells helped us to evaluate the atypia of the clear cells. Diffuse cytoplasmic staining of CEA and CD10 suggested that the clear cell component might harbor malignant potential. We were unable to verify the well-known causes of clear cytoplasm, such as an accumulation of glycogen, lipid, or mucin and enteroblastic differentiation. The causes of clear cells in the colorectal region remain uncertain; however, possible explanations include autolysis and carbohydrate elution.

## Background

In many organs, including the ovary, uterus, kidney, salivary gland, thyroid gland, skin, and breast, when tumor cells show clear cytoplasm, the diagnoses reflect the clear cell morphology, such as clear cell adenocarcinoma (CCA), clear cell carcinoma, glycogen-rich adenocarcinoma, lipid-rich adenocarcinoma, and other clear cell variants of each tumor [1]. These diagnoses tend to be used when the clear cell nature of the tumor is evident [1]. In colorectal tubular adenoma or adenocarcinoma, conversely, clear cells are rarely observed, the reason for which remains uncertain [2–26]. Herein, we report an additional case of tubular adenoma and of tubular adenocarcinoma, both of which have a clear cell component. We describe a thorough investigation of its clear cell etiology and review the literature.

## Case presentation

### Case 1

A 75 years old man with a past history of gastric cancer was introduced to Oita Prefectural Hospital for a routine colonoscopy examination. An 18 × 12 mm superficial elevated polyp was detected in the rectum and resected endoscopically.

**Fig. 1:**
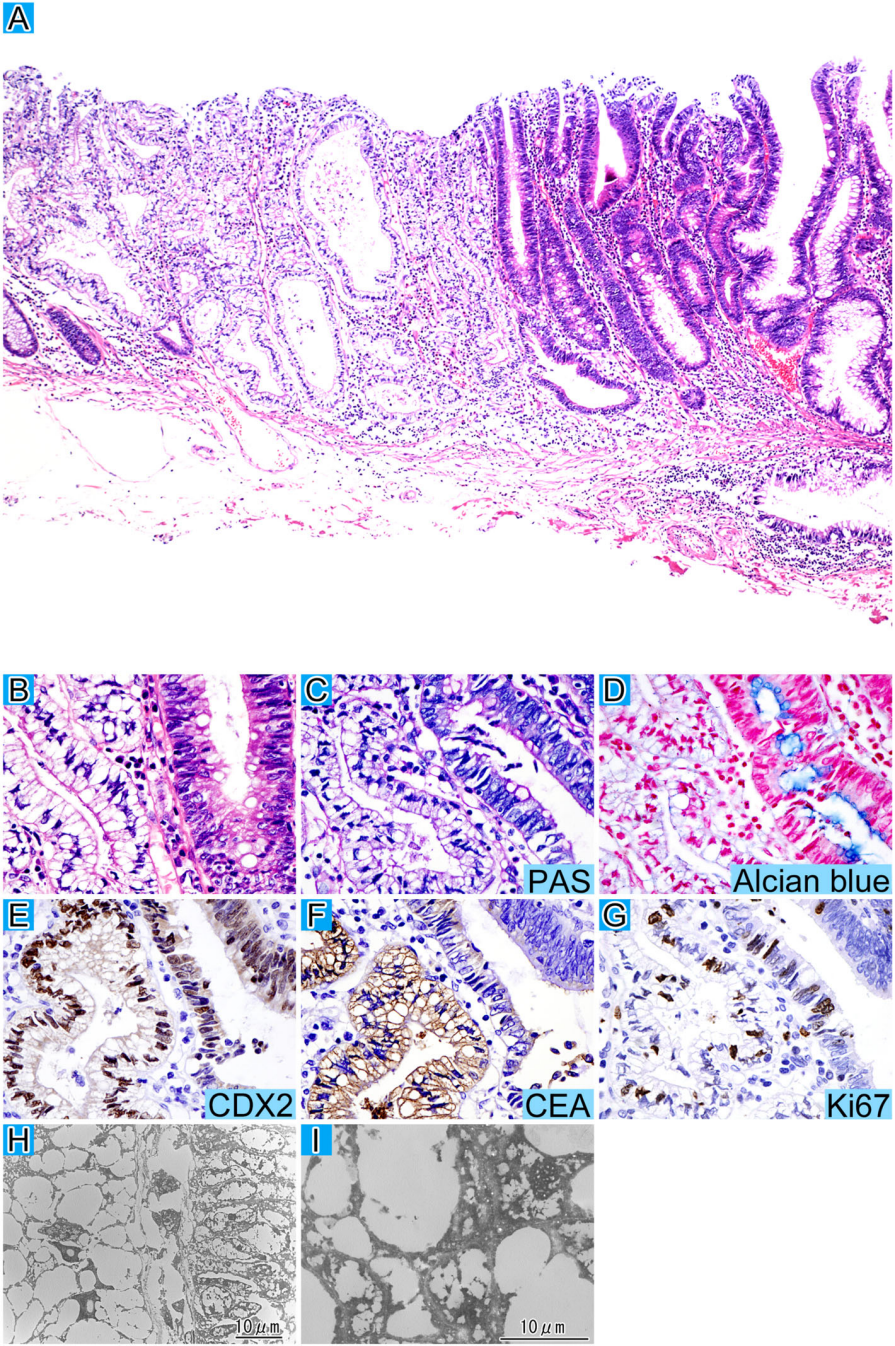
Tubular adenoma with clear cell change. The striking tubule structures of the clear cells are accompanied by conventional tubular adenoma cells at low magnification with HE staining (**a**). The boundary between the clear cell and conventional components at high magnification with HE staining (**b**). The clear cell component is negative for PAS (**c**) and alcian blue staining (**d**). Both components are positive for CDX2 staining (**e**). The localization of CEA (f) expression is diffusely cytoplasmic for the clear cell component, and luminal cell apical for the conventional one. Ki67 labeling (**g**) is slightly lower in the clear cell component. d-g represent immunohistochemistry. Ultrastructural examination (**h**) of the boundary between the clear cell area (left) and the conventional adenoma (right) at low magnification is shown and multiple cytoplasmic lipid-like vacuoles surround the nuclei in the clear cells (**i**)

Microscopically, 90% of the tumor cells showed dysplastic columnar epithelium with hyperchromatic short spindle nuclei regularly arranged in the basal portion and eosinophilic cytoplasm (Fig. 1a and b). We diagnosed it as conventional tubular adenoma with low grade dysplasia. Additionally, 10% of the tumor cells had dysplastic columnar epithelium with randomly arranged pyknotic polygonal nuclei and clear cytoplasm (Fig. 1a and b).

Periodic acid-Schiff (PAS), PAS diastase (PAS-D), Alcian blue, and mucicarmine staining were all negative for the clear cell component (Fig. 1c and d). The antibodies used in this study are listed in Table 1. Immunohistochemically, both tumor components were negative for CK7, focally positive for CK20, and positive for CDX2 (Fig. 1e). A difference in results was observed following staining for carcinoembryonic antigen (CEA) (Fig. 1f). Positive CEA staining was found on the luminal side in the conventional area of the tumor; however, diffuse cytoplasmic staining was observed in the clear cell area. MUC2, MUC5AC, MUC6, CD10, AFP, AR, perilipin, and adipophilin were all negative for clear cell components. The Ki67 (Fig. 1g) labeling index (LI) was 83.7 and 73.8% for conventional and clear cell components, respectively. Electron microscopic examination found multiple lipid-like vacuoles in the clear cell component but not in the conventional component (Fig. 1h and i). He received regular follow-up and did not have a recurrence for 4 years.

**Table 1:** Antibody information

Antibody	Clone	Company	Dilution	Conditioning
CK7	OV-TL 12/30	DAKO, Santa Clara, USA	1:50	Protease
CK20	Ks 20.8	DAKO, Santa Clara, USA	1:50	Protease
CDX2	AMT28	Novus Biologicals, Newcastle, UK	1:50	pH 9.0
CEA	II-7	DAKO, Santa Clara, USA	1:40	pH 6.0
CD10	56C6	Novocastra, Newcastle, UK	1:50	pH 6.0 overnight
MUC2	Ccp58	Novocastra, Newcastle, UK	1:100	pH 6.0
MUC5AC	CLH2	Abcam, Cambridge, UK	1:1000	
MUC6	CLH5	Novocastra, Newcastle, UK	1:100	pH 6.0
AFP	N1501	DAKO, Santa Clara, USA	diluted	
Glypican3	1G12	Nichirei Bioscience, Tokyo, Japan	diluted	pH 9.0
AR	AR441	DAKO, Santa Clara, USA	1:25	pH 6.0
Perilipin	GP29	PROGEN, Heidelberg, Germany	1:200	pH 6.0
Adipophilin	AP125	Acris Antibodies, Herford, Germany	1:10	pH 6.0
Ki67	MIB-1	DAKO, Santa Clara, USA	1:50	pH 6.0
COX2	CX-294	DAKO, Santa Clara, USA	1:100	pH 9.0
APC	CC-1	Oncogene, California, USA	1:20	pH 6.0

*CK* Cytokeratin, *CDX2* Caudal type homeobox 2, *CEA* Carcinoembryonic antigen, *CD* Cluster differentiation, *MUC* Mucin, *AFP* Alpha fetoprotein, *AR* Androgen receptor, COX2 Cyclooxygenase 2, *APC* Adenomatous polyposis coli

### Case 2

**Fig. 2:**
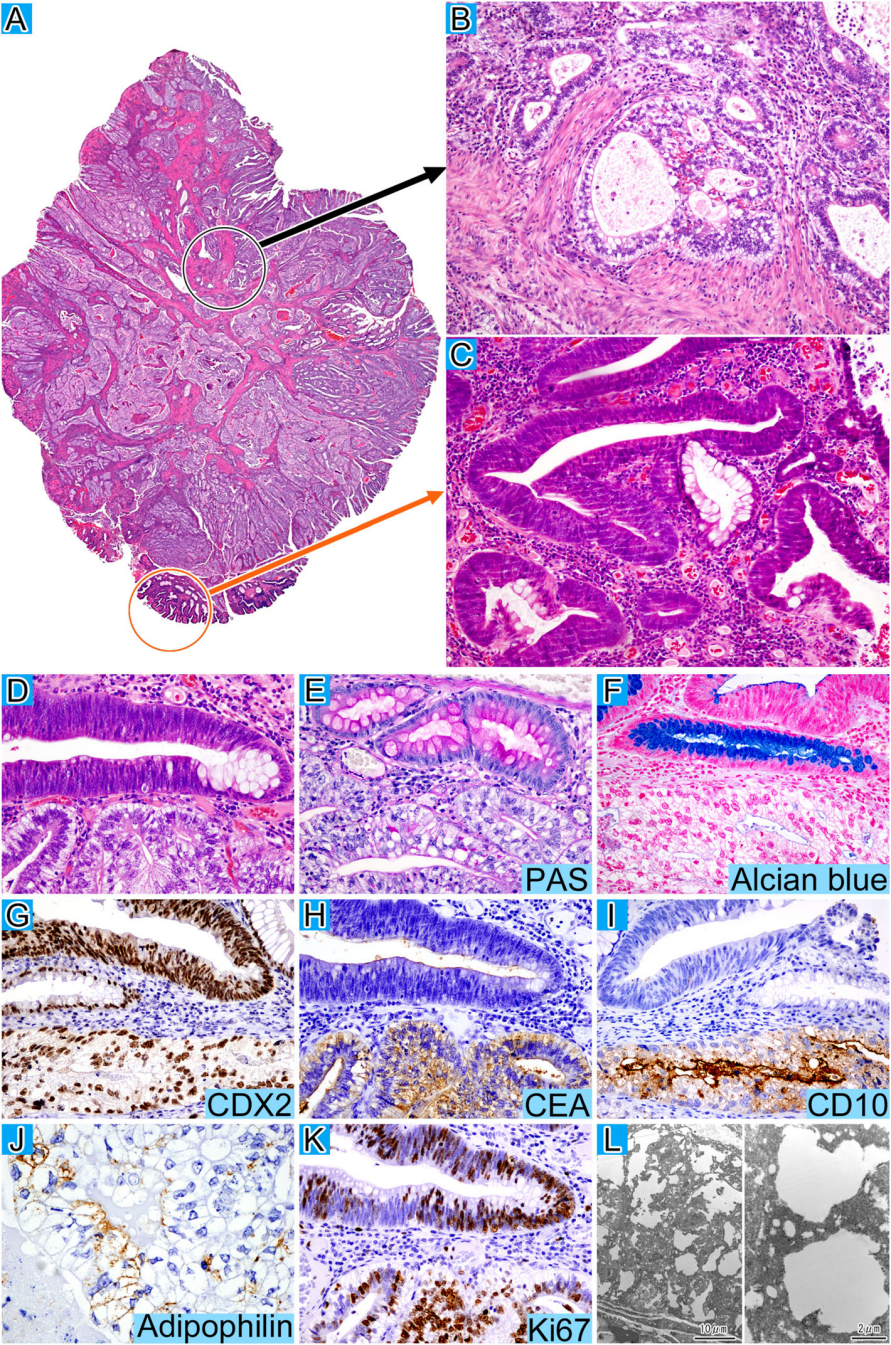
Clear cell adenocarcinoma. Low magnification (**a**) and high magnification of the clear cell (**b**) and conventional components (**c**) with HE staining. The boundary (**d**) between the clear cell and conventional components at high magnification with HE staining. The clear cells are negative for PAS (**e**) and alcian blue staining (**f**), whereas both components of the tumor are positive for CDX2 (**g**). The localization of CEA (**h**) expression is diffusely cytoplasmic for the clear cell component and luminal cell apical for the conventional component. CD10 (**i**) and adipophilin (**j**) expression is confined to the clear component and Ki67 labeling (**k**) is higher in the clear cell component. g-k represent immunohistochemistry. Immunoelectron microscopy analysis (**l**) at low (left) and high magnification (right) reveals multiple cytoplasmic lipid-like vacuoles in clear cells that are negative for adipophilin

A 58-year-old man was admitted to Oita University Hospital for the medical examination of an abnormality. The contrast CT examination showed a wall thickness of the sigmoid colon and a colonoscopy was performed. There were multiple polyps detected in the sigmoid colon and a 25 mm in size pedunculated polyp was endoscopically resected. Microscopically, 10% of the tumor cells were conventional tubular adenocarcinoma with hyperchromatic oval nuclei regularly arranged in the basal portion and eosinophilic cytoplasm (Fig. 2a, b, c, and d). The other tumor cells displayed dysplastic columnar epithelium with large epithelioid or polygonal nuclei randomly arranged and clear or vacuolated cytoplasm, showing cribriform or fused tubular structures and desmoplastic reaction was seen in the surrounding stroma (Fig. 2b). These findings were thought to be invasion. Tumor invaded into submucosa (pT1b).

PAS, PAS-D, Alcian blue, and mucicarmine staining were all negative for the clear cell component (Fig. 2e and f). Immunohistochemically, both tumor components were negative for CK7, focally positive for CK20, and positive for CDX2 (Fig. 2g) and MUC2. The differences in results between the two components were staining for CEA (Fig. 2h) and CD10 (Fig. 2i). Positive CEA staining was observed for the luminal aspect in the conventional component; however, there was diffuse cytoplasmic staining in the clear cell component. CD10 was only positive for the clear cell part and adipophilin (Fig. 2j) was only focally positive for clear cell component. MUC5AC, MUC6, AFP, glypican 3, perilipin, and AR were all negative. COX2 and APC were weakly diffuse cytoplasmic staining for both components, but the staining of APC seemed to attenuate or disappear in invasive areas. The Ki67 LI (Fig. 2k) was 80.0% and almost 100% for conventional and clear cell components, respectively. An immunoelectron microscopic examination was performed according to procedures described in a previous study [27] and showed that the nuclei were surrounded by multiple cytoplasmic lipid-like vacuoles similar to case 1 and they were mostly negative for adipophilin (Fig. 2l). Postoperative follow-up testing such as a laboratory examination, CT imaging, and endoscopic examination didn’t show any sign of recurrence and he was free from the disease for 1 year and a half.

## Discussion and conclusions

A clear cell colorectal tumor was first described by Hellstrom’s report of a physaliferous variant of colon adenocarcinoma [10]. Clear cells were then detected in colorectal tubular adenoma, hyperplastic polyps, and tubular adenocarcinoma [2–9]. Domoto et al. [5] retrospectively analyzed the probability of clear cell tubular adenoma and its incidence was only 0.086%.

To date, there have been 44 cases of clear cell of colorectal epithelial tumors reported, composed of 20 adenomas (Table 2) and 24 adenocarcinomas (Table 3) [2–26]. The median age was 57.2 and 58.6 years for adenoma and adenocarcinoma, respectively. Both tumors showed a male predilection (adenoma: 11/17, adenocarcinoma: 18/23) and occurred mostly in the left-side colon (adenoma: 14/19, adenocarcinoma: 16/24). Some cases had multiple polyps at the same time [3, 7, 9, 11, 14, 16, 20] and two cases had multiple tubular adenomas with a clear cell component [7, 9]. Case 2 reported here had multiple polyps; however, no other polyps had clear cell components.

**Table 2:** Clinicopathological information for 20 colorectal adenomas with clear cell components

Author^Ref^	Age	Sex	Location	Size (cm)	PAS	Alcian blue	Prognosis
Reed et al. [2]	ND	ND	ND	ND	ND	ND	
Jewell et al. [3]	61	F	R	2	–	–	Alive
Suzuki et al. [4]	62	M	D	1.4	–	–	Alive
Domoto et al. [5]	54	M	S	6	–	Scattered	
	45	M	T	6	–	Scattered	
	44	M	S	10	–	Scattered	
Eloy et al. [6]	48	F	T	2.5	ND	–	
	68	F	S	0.5	ND	–	
	84	M	S	1.8	ND	–	
Shi et al. [7]	ND	ND	ND	0.8	ND	ND	
	61	M	S	1.5	ND	ND	
	ND	ND	ND	1.8	ND	ND	
	63	F	As	0.5	–	ND	
	63	M	R	1.4	–	ND	
	68	F	ND	3.5	–	ND	
	30	F	S	1.4	–	ND	
	35	M	S	1.3	ND	ND	
Yao [8]	48	M	S	0.8	–	–	
Miyasaka et al. [9]	63	M	As, D, R	ND	–	–	
Present study	75	M	R	1.8	–	–	Alive

*M* Male, *F* Female, *As* Ascending colon, *T* Transverse colon, *D* Descending colon, *S* Sigmoid colon, *R* Rectum, *ND* No data

**Table 3:** Clinicopathological information for 24 colorectal adenocarcinomas with clear cell components

Author^Ref^	Age	Sex	Location	Size (cm)	PAS	Alcian blue	Prognosis
Hellstrom and Fisher [10]	67	M	R	2	−/+	–	Alive
Reed et al. [2]	71	M	T	7	+		ND
Jewell et al. [3]	75	M	S	0.1	–	–	Died
	56	F	S	6	ND	–	ND
Watson [11]	58	M	AC	3.5	+	–	Died
Rubio [12]	68	M	D	6	−/+	–	Died
Furman and Lauwers [13]	ND	ND	R	ND	+	ND	ND
Braumann et al. [14]	89	M	T	2.2	–	–	Died
Mallik and Katchy [15]	36	F	R	5	+	+	ND
Ko et al. [16]	62	M	S	1.5	ND	ND	ND
Hao et al. [17]	37	M	D	12	+	–	Alive
Barisella et al. [18]	54	M	As	0.9	ND	ND	Alive
Soga et al. [19]	71	F	S	0.8	–	–	ND
Bressenot et al. [20]	84	F	D	3.5	–	–	Alive
Shi et al. [7]	52	M	R	0.9	–	ND	ND
	51	M	S	1.4	–	ND	ND
Furuya et al. [21]	81	M	As	9.5	+	ND	Died
Barrera-Maldonado et al. [22]	41	F	D	3.4	ND	ND	ND
Wang et al. [23]	26	M	T	12	ND	ND	Died
Thelin et al. [24]	25	M	As	3	ND	ND	Died
Remo et al. [25]	58	M	As	7	ND	ND	Died
	79	M	As	4.5	ND	ND	Died
Tochio et al. [26]	48	M	D	0.7	–	–	Alive
This study	58	M	S	2.5	–	–	Alive

*M* Male, *F* Female, *As* Ascending colon, *T* Transverse colon, *D* Descending colon, *S* Sigmoid colon, *R* Rectum, *AC* Anal canal, ND No data

Histologically, the clear cells of colorectal tumors characteristically have pyknotic polygonal nuclei not confined to the basal portion but randomly arranged and clear or vacuolated cytoplasm [5]. In our cases, it was difficult to assess the nuclear atypia of clear cells, however, conventional tubular adenoma or adenocarcinoma cells accompanied them and there was a transition between both components. This helped us to recognize the clear cell components as the atypical equivalent to adenoma or adenocarcinoma. Moreover, it may be misleading to diagnose metastatic carcinoma if the clear cell component accounts for the vast majority of the tumor. Therefore, it is important to confirm a colorectal origin by immunohistochemical analysis of CK7, CK20, and CDX2 [7]. Our cases were CK7 negative, CK20 focally positive, and CDX2 diffusely positive, consistent with a colorectal origin.

Differences in staining results between the conventional and clear cell component were found for CEA and CD10. The localization of CEA is associated with tumor differentiation; thus, luminal cell apical expression of CEA is seen in well-differentiated tumors and, in contrast, cytoplasm expression is seen in poorly differentiated tumors [28]. The tumor cell phenotype correlates with tumor aggressiveness and biological behavior in several cancers. The expression of CD10 suggests colorectal adenocarcinoma with small intestinal differentiation, which is associated with higher venous invasion than large intestinal phenotype of colorectal adenocarcinoma [29]. The diffuse cytoplasmic expression of CEA and the confined expression of CD10 seen in clear cell areas may indicate that these clear cell components harbor greater malignant potential.

Generally, an accumulation of glycogen, mucin, and lipid, as well as enteroblastic differentiation, are well-known examples of the etiology or substances of clear cells in HE specimens. Colorectal tubular adenoma and adenocarcinoma with clear cell components were reported as tubular adenoma with clear cell change (TAC) and CCA, respectively [2–26]. In TAC, an accumulation of glycogen, mucin, and enteroblastic differentiation have never been verified by PAS, Alcian blue staining, or AFP immunostaining [2–9]. Case 1 is negative for those stains, in accord with previous TAC reports. In CCA, on the other hand, some cases are either positive for PAS [2, 11, 13, 15, 17, 21], Alcian blue staining [15], or AFP immunohistochemistry [21]; however, other cases are negative for these stains [3, 10, 12, 14, 19, 20, 26]. It seems that some heterogeneity exists among CCA cases [2, 3, 7, 10–26]. Case 2 is negative for PAS, Alcian blue staining, and AFP immunostaining and corresponds to previously reported negative-result cases. The pathogenesis of TAC and some CCA cases, including our cases, remains unclear. It may be more appropriate to diagnose Case 2 and those previously reported negative-result cases as tubular adenocarcinoma with clear cell change, corresponding with the malignant counter part of TAC, rather than CCA.

Electron microscopic examination revealed multiple cytoplasmic lipid-like vacuoles in the clear cells of both cases, and this finding corresponded to previous reports [3, 5, 10–12]. These vacuoles were described as autolysis or elution of glycogen granules during processing or fixation [5, 10], glycogen-like material with lipid to a lesser extent [11], and degeneration due to lipid accumulation [6, 9]. Miyasaka et al. [9] recently reported one case of TAC positive for adipophilin immunostaining and described that lipid accumulation might be responsible for its clear cell nature. In our report, Case 1 was negative for adipophilin but case 2 showed focal positive staining for it, which caused us to consider lipid accumulation; however, the immunoelectoron microscopy results were mostly negative for adipophilin. Bressenot et al. [20] reported a CCA and described that they could not detect glycogen in formalin sections but found it in frozen sections. In our study, we could not determine what caused the clear cells in the colorectal tumors; however, autolysis or carbohydrate elution remain possible explanations.

## Conclusions

We report two colorectal tumors with a clear cell component. Accompanying components of conventional tubular adenoma or adenocarcinoma helped us to evaluate the atypia of the clear cells. Differences in staining results for CEA and CD10 were observed and the clear cell component might harbor malignant potential. We tested for the well-known causes of clear cells; however, none were detected. An electron microscopic examination found multiple cytoplasmic lipid-like vacuoles in the clear cell component; however, they were largely negative for adipophilin by immunoelectron microscopy. The causes of clear cells in the colorectal region remain uncertain.

## Abbreviations

CCA: Clear cell adenocarcinoma
CEA: carcinoembryonic antigen
LI: Labeling index
PAS: Periodic acid-Schiff
PAS-D: PAS diastase
TAC: Tubular adenoma with clear cell change
